# Prospective Validation of FibroTest in Comparison with Liver Stiffness for Predicting Liver Fibrosis in Asian Subjects with Chronic Hepatitis B

**DOI:** 10.1371/journal.pone.0035825

**Published:** 2012-04-20

**Authors:** Beom Kyung Kim, Seung Up Kim, Hyon Suk Kim, Jun Yong Park, Sang Hoon Ahn, Chae Yoon Chon, In Rae Cho, Dong-Hoo Joh, Young Nyun Park, Kwang-Hyub Han, Do Young Kim

**Affiliations:** 1 Department of Internal Medicine, Yonsei University College of Medicine, Seoul, Korea; 2 Yonsei Institute of Gastroenterology, Yonsei University College of Medicine, Seoul, Korea; 3 Department of Laboratory Medicine, Yonsei University College of Medicine, Seoul, Korea; 4 Department of Pathology, Yonsei University College of Medicine, Seoul, Korea; 5 Liver Cirrhosis Clinical Research Center, Yonsei University College of Medicine, Seoul, Korea; 6 Brain Korea 21 Project for Medical Science, Yonsei University College of Medicine, Seoul, Korea; The University of Hong Kong, Hong Kong

## Abstract

**Background and Aims:**

Diagnostic values of FibroTest (FT) for hepatic fibrosis have rarely been assessed in Asian chronic hepatitis B (CHB) patients. We aimed to validate its diagnostic performances in comparison with liver stiffness (LS).

**Methods:**

From 2008 to 2010, 194 CHB patients who underwent liver biopsies along with FT and transient elastography were prospectively enrolled. Fibrosis stage was assessed according to the Batts and Ludwig system.

**Results:**

To predict significant fibrosis (F≥2), advanced fibrosis (F≥3), and cirrhosis (F = 4), areas under receiver operating characteristic curves (AUROCs) of FT were 0.903, 0.907, and 0.866, comparable to those of LS (0.873, 0.897, and 0.910, respectively). Optimized cutoffs of FT to maximize sum of sensitivity and specificity were 0.32, 0.52, and 0.68 for F≥2, F≥3, and F = 4, while those of LS were 8.8, 10.2, and 14.1 kPa, respectively. According to FT and LS cutoffs, 123 (63.4%) and 124 (63.9%) patients were correctly classified consistent with histological fibrosis (F1, F2, F3, and F4), respectively. Overall concordance between each fibrosis stage estimated by FT and LS was observed in 111 patients, where 88 were correctly classified with histological results. A combination formula adding LS to FT (LS+FT) showed similar AUROC levels (0.885, 0.905, and 0.915), while another multiplying LS by FT (LS×FT) showed the best AUROCs (0.941, 0.931, and 0.929 for F≥2, F≥3, and F4, respectively).

**Conclusions:**

FT provides good fibrosis prediction, with comparable outcomes to LS in Asian CHB patients. FT substantially reduces need for liver biopsy, especially when used in combination with LS.

## Introduction

Accurate assessment of the severity of liver fibrosis in patients with chronic hepatitis B (CHB) is necessary not only for predicting the long-term clinical course, but also for determining whether and when to begin antiviral therapy. Most recent guidelines on the management of CHB have proposed that the presence of significant fibrosis with detection of serum hepatitis B virus (HBV) DNA is a clear indication to commence antiviral therapy, because maintenance of viral suppression can reduce liver-related complications in patients with CHB who have significant fibrosis or cirrhosis [1], [2], [3]. Conversely, the absence of significant fibrosis in patients with low levels of circulating virus is an indication to monitor rather than initiate expensive and potentially long-lasting antiviral therapy. Furthermore, as patients with cirrhosis should be followed-up closely for the development of hepatocellular carcinoma and other complications associated with hepatic decompensation, including gastroesophageal varices, assessment of fibrotic burden in patients with CHB has become an important clinical issue for physicians [1], [4].

To date, liver biopsy has been the gold standard for assessing liver fibrosis. It is often limited, however, by its invasiveness, cost, risk of complications, poor acceptance, lack of availability of expert practitioners, and intra/inter-observer variability [5], [6]. These drawbacks make sequential liver biopsies unfeasible, especially when repeated examinations are required to monitor the response to antiviral or antifibrosis treatment. Consequently, these limitations have stimulated the researches for noninvasive approaches, such as the aspartate aminotransferase (AST)-to-platelet ratio index (APRI) [7], AST-alanine aminotransferase (ALT) ratio [8], Forns test [9], and FibroTest (FT; BioPredictive, Paris, France) [10], all of which combine several biochemical parameters [11], [12], [13].

Recently, liver stiffness (LS) value assessed by transient elastography (TE; FibroScan®; Echosens, Paris, France), which relies on calculating liver elasticity from the velocity of a low frequency elastic wave transmitted through the liver, has been introduced as a noninvasive surrogate for liver biopsy in the assessment of liver fibrosis [14], [15], [16]. As TE was first developed in France, most studies on its benefits have been performed in European countries where chronic hepatitis C (CHC) is prevalent. However, due to vigorous efforts to apply TE to Asian subjects with CHB, it has now been shown to have acceptable accuracy in diagnosing liver fibrosis and cirrhosis in these subjects [17].

Meanwhile, Poynard *et al.* [10] in 2001, proposed a scoring algorithm using a panel of five biochemical markers, i.e., FT, including α_2_-macroglobulin, apolipoproteinA1, haptoglobin, γ-glutamyl-transpeptidase (GGT), and total bilirubin for assessment of liver fibrosis, adjusted by age and gender. FT has been studied extensively as a surrogate marker for liver biopsy. It was initially validated primarily in Caucasian populations with CHC and the results showed a good correlation with liver fibrosis stage [18], [19], [20]. However, in contrast to TE, only a few studies have been reported to date in patients with CHB [21], [22], [23], [24], [25]. In particular, the investigation focusing Asian population with CHB has been extremely scarce.

The present study was performed to prospectively validate the diagnostic value of FT in Asian populations with CHB in comparison with TE, to define optimized thresholds for predicting liver fibrosis, and to investigate the performance of the combined use of FT and LS.

## Materials and Methods

### 1. Patients

Consecutive patients with CHB who underwent liver biopsy along with FT and TE on the same day at Severance Hospital, Yonsei University College of Medicine, Seoul, Korea, between July 2008 and June 2010, were considered eligible for this study. Liver biopsy was performed to assess the severity of fibrosis and inflammation prior to treatment.

The exclusion criteria were as follows: previous history of antiviral therapy; history of hepatocellular carcinoma (HCC) treatment at the time of liver biopsy; diagnosis of malignancy other than HCC during follow-up; liver biopsy specimen shorter than 20 mm; coinfection with human immunodeficiency virus; invalid LS values with fewer than ten successful acquisitions, a success rate of less than 60%, or interquartile range (IQR)/median value ratio (IQR/M) greater than 0.3; alcohol ingestion in excess of 40 g/day for more than 5 years; or right-sided heart failure.

The study was performed in accordance with the ethical guidelines of the 1975 Declaration of Helsinki. Written informed consent was obtained from each participant or responsible family member after possible complications of the diagnostic procedures had been fully explained. This study was approved by the Institutional Review Board of Severance Hospital.

### 2. Serum biochemical tests and FT score calculation

All laboratory data including specific parameters for calculating FT score including α2-macroglobulin level, apolipoprotein A1 level, haptoglobin level, γ-GGT level, and total bilirubin level were obtained on the same day as TE and liver biopsy. FT score was computed on the BioPredictive website (www.biopredictive.com) as follows: f = 4.467×log[α2-macroglobulin (g/L)]−1.357×log[haptoglobin (g/L)]+1.017×log[γ-GGT (IU/L)]+0.0281×[age (in years)]+1.737×log[bilirubin (µmol/L)]−1.184×[apolipoprotein A1 (g/L)]+0.301×sex (female = 0, male = 1)−5.540.

### 3. Assessment of LS values

TE was performed by one well-trained technician using FibroScan® on the same day as FT. Details of the technique and examination procedure were reported previously [26], [27]. The results were expressed in kilopascals (kPa). IQR was defined as an index of intrinsic variability of LS values corresponding to the interval of LS results containing 50% of the valid measurements between the 25^th^ and 75^th^ percentiles. The median value was considered representative of the elastic modulus of the liver. Only procedures with at least 10 valid measurements, a success rate of at least 60%, and an IQR-to-median ratio <30% were considered reliable.

The TE operator was blinded to patients' clinical and laboratory data.

### 4. Liver biopsy examination

Percutaneous liver biopsy was performed using a 16G disposable needle immediately after TE. The liver biopsy specimens were fixed in formalin and embedded in paraffin. Then, sections 4 µm thick were stained with hematoxylin and eosin (H&E) and Masson's trichrome. All liver tissue samples were evaluated by an experienced hepatopathologist (YN Park) who was blinded to the patients' clinical histories. Specimens that were shorter than 20 mm and considered by the pathologists to be unsuitable for fibrosis assessment were excluded from the analysis. Liver histology was evaluated semiquantitatively according to the Batts and Ludwig scoring system [28]. Fibrosis was staged on a 0–4 scale: F0, no fibrosis; F1, portal fibrosis; F2, periportal fibrosis; F3, septal fibrosis; and F4, cirrhosis. Significant fibrosis was defined as F2 or more, advanced fibrosis as F3 or more, and cirrhosis as F4.

### 5. Statistical analyses

The major goals of this study were to prospectively validate the diagnostic performance of FT for detection of the presence of histological significant fibrosis, advanced fibrosis, and cirrhosis in comparison with LS and to suggest optimal cutoff values in patients with CHB. To assess the diagnostic performance of each noninvasive index, receiver operating characteristic (ROC) curves were constructed and the areas under the ROC curves (AUROCs) were calculated. Then, to evaluate the usefulness of the noninvasive method, the sensitivity, specificity, positive predictive value (PPV), and negative predictive value (NPV) were determined from the ROC curves. Furthermore, we evaluated the usefulness of combined use of FT and LS as a surrogate marker for liver biopsy.

Statistical analyses were performed using SAS software version 9.1.3 (SAS, Cary, NC). In all analyses, *p*<0.05 was taken to indicate statistical significance.

## Results

### 1. Patients baseline characteristics

**Figure 1:**
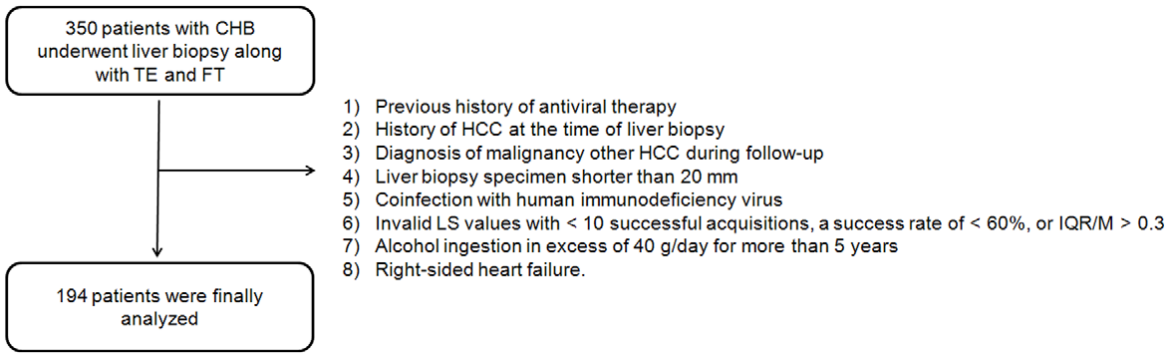
Flow chart describing the selection of the study population. Based on the exclusion criteria, 194 subjects were finally recruited for analyses.

A total of 350 consecutive patients were screened for possible inclusion in the study. Based on the exclusion criteria, a total of 194 patients (mean age 46.7 years, 119 male) were analyzed (Figure 1).

The patients' characteristics are summarized in Table 1. The mean ALT level was 58.4 IU/L, while the mean AST level was 44.1 IU/L. The mean LS and FT were 14.2 kPa and 0.53, respectively. The mean length and the median number of fragments of liver biopsy samples were 21.3 mm and 2, respectively. The fibrosis stages were F0 in 0 (0%) patients, F1 in 30 (15.5%), F2 in 50 (25.7%), F3 in 39 (20.1%), and F4 in 75 (38.7%). All patients had well-preserved liver functions.

**Table 1:** Baseline characteristics (n = 194).

Characteristics	Value
**Demographic data**	
Age (years)	46.7±14.7
Male gender, no. (%)	119 (61.3)
Body mass index (kg/m^2^)	23.4±2.8
**Laboratory data**	
Serum albumin (g/dL)	4.75±1.37
Total bilirubin (mg/dL)	1.16±0.90
Aspartate aminotransferase (IU/L)	44.1±28.3
Alanine aminotransferase (IU/L)	58.4±27.1
Prothrombin time (%)	93.1±13.3
Platelet count (10^9^/L)	179.3±71.2
**Biopsy length (mm)**	21.3±0.7
**Liver stiffness (kPa)**	14.2±9.5
**FibroTest**	0.53±0.29
**Fibrosis stage, no. (%)**	
F0	0 (0)
F1	30 (15.5)
F2	50 (25.7)
F3	39 (20.1)
F4	75 (38.7)

Values were expressed as mean ± standard deviation, unless indicated otherwise.

### 2. Diagnostic performances of LS and FT

**Figure 2:**
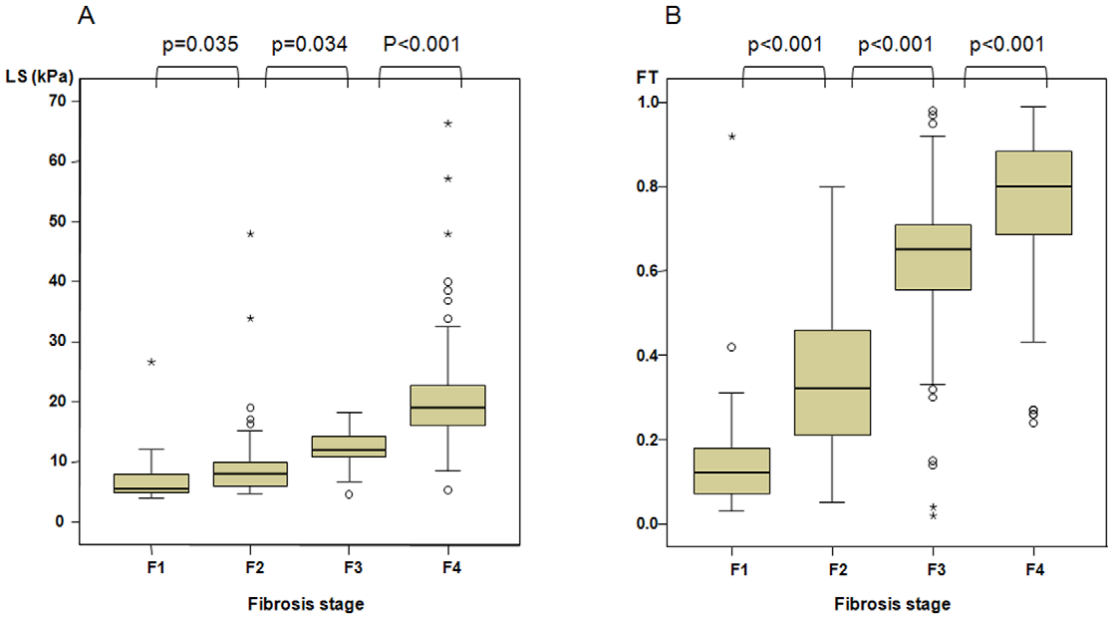
Box plots of LS (A) and FT (B) according to fibrosis stage. Boxes and horizontal lines within boxes represent interquartile ranges (IQRs) and median values, respectively. The upper and lower whiskers indicate 75^th^ percentile plus 1.5 IQR and 25^th^ percentile minus 1.5 IQR, respectively. o, mild outlier: a value more than 75^th^ percentile plus 1.5 IQR, but less than 75^th^ percentile plus 3.0 IQR. *, extreme outlier: a value more than 75^th^ percentile plus 3 IQR.

As shown in Figure 2, the overall mean values of LS (Fig. 2A) and FT (Fig. 2B) increased in parallel with the increase in fibrosis stage (all *p*<0.05). As the fibrosis stage increased from F1 to F4, the mean value of LS increased from 6.9±4.2 kPa in F1, 9.7±5.7 kPa in F2, 12.1±2.97 kPa in F3, and 21.1±10.1 kPa in F4, while that of FT also increased from 0.16±0.17 in F1, 0.34±0.18 in F2, 0.6±0.23 in F3, and 0.76±0.19 in F4. LS and FT were significantly different between F1 and F2 (*p* = 0.035 and *p*<0.001), F2 vs. F3 (*p* = 0.034 and *p*<0.001), and F3 vs. F4 (*p*<0.001 and *p*<0.001), respectively.

**Figure 3:**
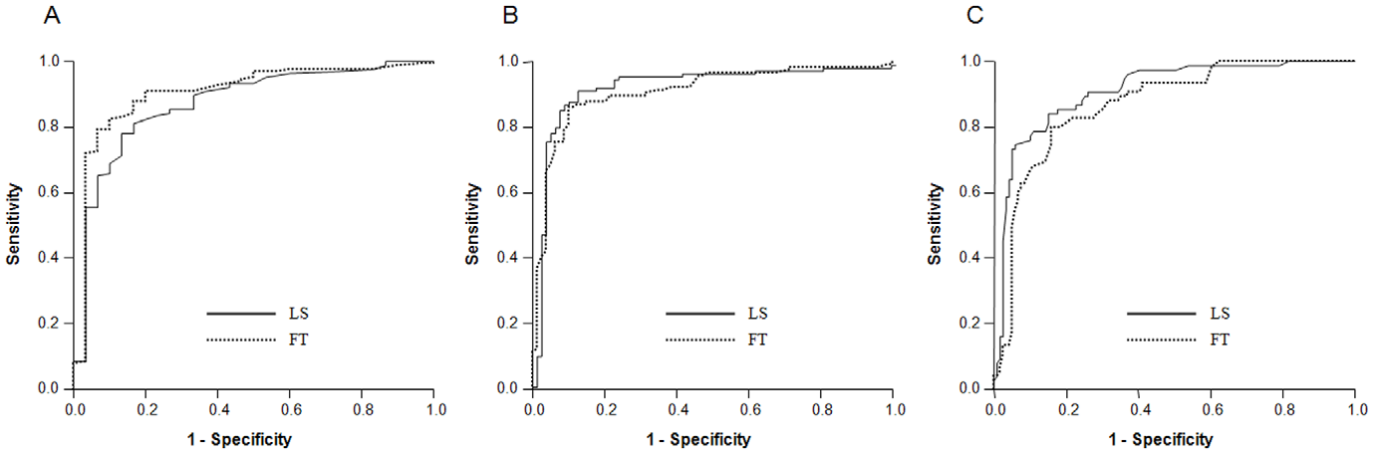
Receiver operating characteristic (ROC) curves for LS and FT in the diagnosis of significant fibrosis (≥F2, A), advanced fibrosis (≥F3, B), and cirrhosis (F = 4, C).

With regard to the diagnostic performances of LS and FT in the prediction of histological liver fibrosis, the AUROCs of LS and FT were 0.873 (95% confidence interval [CI] 0.802–0.944) and 0.903 (95% CI 0.838–0.968) for significant fibrosis (F≥2) (Fig. 3A), 0.897 (95% CI 0.846–0.949) and 0.907 (95% CI 0.862–0.952) for advanced fibrosis (F≥3) (Fig. 3B), and 0.910 (95% CI 0.867–0.953) and 0.866 (0.815–0.918) for cirrhosis (F = 4) (Fig. 3C), respectively (Table 2). There were no significant differences between AUROC values of LS and FT (all *p*>0.05 by Hanley and McNeil test) [29].

**Table 2:** Diagnostic performances of LS and FT and their suggested optimal cutoff values.

Method	Fibrosis stage	AUROC (95% CI)	Cutoffs	Sensitivity (%)	Specificity (%)	NPV (%)	PPV (%)
**LS**	**F≥2**	0.873 (0.802–0.944)	8.8 kPa	78.0	86.7	41.9	97.0
	**F≥3**	0.897 (0.846–0.949)	10.2 kPa	86.3	90.4	86.3	90.4
	**F = 4**	0.910 (0.867–0.953)	14.1 kPa	84.0	84.9	89.4	77.8
**FT**	**F≥2**	0.903 (0.838–0.968)	0.32	79.3	93.3	45.2	98.5
	**F≥3**	0.907 (0.862–0.952)	0.52	86.0	90.0	81.8	92.5
	**F = 4**	0.866 (0.815–0.918)	0.68	80.0	84.0	87.0	75.9

LS, liver stiffness; FT, FibroTest; AUROC, area under the receiver operating characteristics curve; CI, confidence interval; NPV, negative predictive value; PPV, positive predictive value.

### 3. Determination of the optimal cutoffs for LS and FT

The most discriminant cutoff values for LS and FT were determined from the ROC curves to maximize the sum of sensitivity and specificity [30] (Table 2). LS cutoff values of 8.8, 10.2, and 14.1 kPa generated sensitivity of 78.0%, specificity of 86.7%, NPV of 41.9%, and PPV of 97.0% for F≥2; sensitivity of 86.3%, specificity of 90.4%, NPV of 86.3%, and PPV of 90.4% for F≥3; and sensitivity of 84.0%, specificity of 84.9%, NPV of 89.4%, and PPV of 77.8% for F = 4, respectively. Similarly, FT cutoff scores of 0.32, 0.52, and 0.68 generated sensitivity of 79.3%, specificity of 93.3%, NPV of 45.2%, and PPV of 98.5% for F≥2; sensitivity of 86.0%, specificity of 90.0%, NPV of 81.8%, and PPV of 92.5% for F≥3; and sensitivity of 80.0%, specificity of 84.0%, NPV of 87.0%, and PPV of 75.9% for F = 4, respectively.

LS agreed with liver biopsy on the diagnosis of F<2 vs. F≥2 in 154 patients (79.3%), F<3 vs. F≥3 in 172 patients (88.7%), and F<4 vs. F = 4 in 164 patients (84.5%), while FT agreed on the diagnosis of F<2 vs. F≥2 in 158 patients (81.4%), F<3 vs. F≥3 in 170 patients (87.6%), and F<4 vs. F = 4 in 160 patients (82.5%) (Table 3). In addition, when using the suggested cutoffs of LS and FT to diagnose each histological fibrosis stage (F1, F2, F3, and F4), 124 (63.9%) and 123 (63.4%) patients (gray-colored area in Table 3) were correctly classified consistent with liver biopsy examination, respectively, and they could avoid liver biopsy according to the corresponding results by noninvasive methods (Table 3).

**Table 3:** Distribution and agreement of fibrosis stages according to histology and LS or FT (n = 194).

	Total	Fibrosis stage estimated by LS				Fibrosis stage estimated by FT			
Fibrosis stage estimated by histology		F1	F2	F3	F4	F1	F2	F3	F4
		LS<8.8 kPa	8.8≤LS<10.2 kPa	10.2≤LS<14.1 kPa	LS≥14.1 kPa	FT<0.32	0.32≤FT<0.52	0.52≤FT<0.68	FT≥0.68
**F1**	30	26	1	2	1	28	1	0	1
**F2**	50	29	13	1	7	24	19	5	2
**F3**	39	5	2	22	10	5	2	16	16
**F4**	75	2	2	8	63	5	4	6	60
Total	194	62	18	33	81	62	26	27	79

LS, liver stiffness; FT, FibroTest.

### 4. Agreement between LS and FT

Irrespective of matching with histological fibrosis stages, 152 patients (78.3%) showed agreement in fibrosis staging using LS and FT for noninvasive estimation of F<2 vs. F≥2, 158 patients (81.4%) for F<3 vs. F≥3, and 146 patients (75.3%) for F<4 vs. F = 4. Thus, when LS and FT agreed for noninvasive prediction of F<2 vs. F≥2, F<3 vs. F≥3, and F<4 vs. F = 4, these results agreed with those of liver biopsy examination in 88.8% (134 of 152 patients), 96.8% (153 of 158 patients), and 94.5% (138 of 146 patients), respectively.

Overall agreement between each fibrosis stage (F1, F2, F3, and F4) estimated by LS and FT was observed in 111 patients. Among them, 88 patients (79.3%) were correctly classified with reference to liver biopsy examinations, and they could avoid liver biopsy based upon concordant results between the two tests (gray-colored area in Table 4).

**Table 4:** Agreements of histological fibrosis stages among patients who showed concordant results between LS and FT (n = 111).

	Total	Fibrosis stage estimated by concordant LS and FT results			
		F1	F2	F3	F4
Fibrosis stage estimated by histology		LS<8.8 kPa	8.8 kPa≤LS<10.2 kPa	10.2 kPa≤LS<14.1 kPa	LS≥14.1 kPa
		& FT<0.32	& 0.32≤FT<0.52	& 0.52≤FT<0.68	& FT≥0.68
F1	24	24	0	0	0
F2	21	16	3	1	1
F3	11	1	0	8	2
F4	55	0	0	2	53
Total	111	41	3	11	56

LS, liver stiffness; FT, FibroTest.

### 5. Discordance between LS and FT

Discordant results between fibrosis stages estimated by LS and FT were identified in 83 patients (42.8%). On multivariate analysis, only the presence of histological cirrhosis was identified as a single significant factor, which showed a negative association with discordance between LS and FT (*p* = 0.009; odds ratio 0.151, 95% CI 0.036–0.628). Among these patients with discordance between LS and FT, 41 and 42 patients showed higher fibrosis stage by LS and by FT, respectively. The baseline characteristics were compared between these two groups, and none was identified as a significant factor capable of explaining this difference.

### 6. Combined use of LS and FT

Based on a previous report by Castéra et al. [31], we examined the diagnostic performance of a combination formula adding LS to FT (LS+FT). It showed a non-significant trend toward better AUROC than LS, but was worse than FT in prediction of F≥2 (0.885, 95% CI 0.816–0.953) and F≥3 (0.905, 95% CI 0.856–0.955). With regard to prediction of cirrhosis, the combination formula showed better AUROC (0.915, 95% CI 0.874–0.956) than LS and FT alone.

Furthermore, we examined the diagnostic performance of another combination formula multiplying LS by FT (LS×FT), which showed the best AUROC for prediction of F≥2 (0.941, 95% CI 0.908–0.975), F≥3 (0.931, 95% CI 0.889–0.974), and F = 4 (0.929, 95% CI 0.894–0.965), compared to LS, FT, and LS+FT. The optimized cutoff values of combination formula, LS+FT and LS×FT, are described in detail in Table 5.

**Table 5:** Diagnostic performances of combination formula using LS and FT and their suggested optimal cutoff values.

Method	Fibrosis stage	AUROC (95% CI)	Cutoffs	Sensitivity (%)	Specificity (%)	NPV (%)	PPV (%)
**LS+FT**	**F≥2**	0.885 (0.816–0.953)	8.2	84.8	83.3	50.0	96.5
	**F≥3**	0.905 (0.856–0.955)	10.7	93.0	87.5	89.7	91.4
	**F = 4**	0.915 (0.874–0.956)	16.8	76.0	94.1	86.2	89.1
**LS×FT**	**F≥2**	0.941 (0.908–0.975)	2.3	82.9	96.7	50.9	99.3
	**F≥3**	0.931 (0.889–0.974)	4.7	92.1	87.5	88.6	91.3
	**F = 4**	0.929 (0.894–0.965)	9.8	80.0	92.4	88.0	87.0

LS, liver stiffness; FT, FibroTest; AUROC, area under the receiver operating characteristics curve; CI, confidence interval; NPV, negative predictive value; PPV, positive predictive value.

Using the above cutoffs, LS+FT agreed with liver biopsy on the diagnosis of F<2 vs. F≥2 in 164 patients (84.5%), F<3 vs. F≥3 in 176 patients (90.7%), and F<4 vs. F = 4 in 169 patients (87.1%), while LS×FT agreed on the diagnosis of F<2 vs. F≥2 in 165 patients (85.1%), F<3 vs. F≥3 in 175 patients (90.2%), and F<4 vs. F = 4 in 170 patients (87.6%). Overall, 129 (66.5%) and 130 (67.0%) patients were correctly classified consistent with each histological fibrosis stage (F1, F2, F3, and F4) using LS+FT and LS×FT, respectively.

## Discussion

FT, a good surrogate marker for liver biopsy, has been widely studied regarding its usefulness for noninvasive prediction of fibrosis stage primarily in western populations with HCV infection [10]. However, only a few investigations have been conducted in populations with CHB [21], [22], [23], [24], [25]. To the best of our knowledge, we are the first to assess the diagnostic value of FT and to define new cutoff values for each fibrosis stage optimized for a homogenous Asian population with CHB. As there may be a varied spectrum in diagnostic cutoffs of such noninvasive indices based on biochemical parameters, even among studies in patients with the same etiology, a new study to generate standardized, generalized results in Asian patients with CHB is warranted.

Although the underlying mechanisms of fibrosis progression in chronic viral hepatitis are expected to be similar, several differences according to etiology may affect diagnostic accuracy [32], [33]. For example, patients with CHC often have steatosis, which may influence baseline biochemical parameters, and have micronodular cirrhosis. Those with CHB more frequently experience a wide range of fluctuations in necroinflammatory activity and have macronodular cirrhosis leading to relatively lower fibrotic contents than those with CHC [33]. These clinicopathological differences have been suggested to partially explain the relatively lower cutoff LS values in patients with CHB than in those with CHC [27]. Hence, in the present study recruiting Asian patients primarily with CHB, we investigated the accuracy and applicability of FT, which is the most accurate index among patients with CHC.

This study has several strengths. First, we prospectively recruited patients who underwent the baseline blood tests and LS on the same day as liver biopsy, and the diagnostic performance of FT was compared to LS, which has already shown excellent diagnostic value in Asian populations with CHB [26], [34], [35], [36]. Furthermore, a relatively large number of subjects from a single center were consecutively enrolled in this study, and the distribution of our population was homogeneous and representative of patients with CHB seen in clinical practice. Therefore, the optimal cutoff values of FT derived from our study are ultimately expected to be used as reference values for future studies to elaborate on the role of FT in Asian patients with CHB.

In the present study, the diagnostic performance of FT was comparable to that of LS for diagnosing fibrosis stages: 0.903 vs. 0.873 for significant fibrosis (F≥2), 0.907 vs. 0.897 for advanced fibrosis (F≥3), and 0.866 vs. 0.910 for cirrhosis (F = 4), respectively. Using the Youden method [30], we suggested FT cutoff values of 0.32, 0.52, and 0.68 for F≥2, F≥3, and F = 4, respectively. All of these values were slightly lower than the suggested cutoff values for CHB by BioPredictive (0.49, 0.59, and 0.75, respectively). Although several studies have investigated FT in patients with CHB [21], [22], [23], [24], [25], the optimal cutoffs for each fibrosis stage have not been proposed. Thus, another external validation is required for our new thresholds for Asian CHB subjects.

Among subjects where LS and FT agreed with prediction of F≥2, F≥3, and F = 4 regardless of matching with histological examinations, concordance with liver biopsy examination was observed in 88.8%, 96.8%, and 94.5%, respectively. Furthermore, overall agreement between each fibrosis stage (F1, F2, F3, and F4) estimated by LS and FT was observed in 111 patients (57.2%). Among them, 88 patients (79.3%) were correctly classified with reference to liver biopsy examinations, and they could avoid liver biopsy based on the concordant results between the two tests (Table 4). In contrast to other studies, we demonstrated the concordance between two tests for noninvasive prediction of each fibrosis stage (F1, F2, F3, and F4) along with F≥2, F≥3, and F = 4. Such a high level of diagnostic accuracy consistent with previous investigations is the reason why many experts recommend that liver biopsy should be avoided for those with concordance between two noninvasive tests [20], [31]. In fact, based on these advantages of LS and FT, these two markers have been used as the first-line estimates of fibrosis in France instead of liver biopsy [37]. However, for noninvasive prediction of each fibrosis stage, 83 patients (42.8%) had discordant results between LS and FT, and liver biopsy should still be strongly considered in such cases, although several investigators have recently pointed out that the liver biopsy examination has an error rate of up to 20% in disease staging even when an experienced physician performs the liver biopsy examination and an expert pathologist interprets the results [38], [39]. One of the most important methods to minimize such inherent limitations of liver biopsy examination is to obtain the reliable biopsy specimens of adequate sizes [40]. From this viewpoint, we only enrolled patients with so-called reliable biopsy specimens (≥20 mm size) from the beginning of the study.

With regard to discordance between LS and FT, among various baseline factors, only the presence of histological cirrhosis was identified as a single significant factor with a negative association with such discrepancies. Consistent with a previous study [41], patients with histological cirrhosis have a higher rate of non-discordance (84.0%) between LS and histology than those without histological cirrhosis (51.3%, p<0.001). The similar result was maintained for FT in our study (non-discordance of 80.0% in patient without histological cirrhosis vs. 47.1%, in subjects with histological cirrhosis, p<0.001). This negative correlation between the presence of histological cirrhosis and discordances in the present study can be explained in part by the different level of ALT in patients with and without histological cirrhosis (mean value 43.2 vs. 68.1 IU/L, respectively; *p* = 0.008). Because the higher ALT has been known as a well-known overestimating confounder of LS [42] and the same phenomenon was observed for FT in our cohort (p = 0.018) by linear regression analysis with adjusting other variables, patients without cirrhosis were more likely to have discordance between LS (or FT) and liver biopsy. Accordingly, patients with histological cirrhosis and with relatively lower ALT level can have a greater likelihood of non-discordance between LS and FT by diminishing the influence of ALT on LS or FT. However, further validation studies are required to elucidate this issue.

Several investigators [25], [31] tested the combination formula with addition of LS to FT (LS+FT), suggesting that it is likely that a combination of serum biomarkers and LS will complement each other and enhance accuracy of fibrosis detection. However, this method did not always show definitely superior results. In the present study, LS+FT had better AUROC than LS, but was poorer than FT in prediction of F≥2 and F≥3, while it had better AUROCs than LS or FT alone in prediction of F = 4. Next, we assessed the diagnostic value of LS×FT, which consistently showed better AUROCs than LS or FT alone in prediction of F≥2, F≥3, and F = 4. When compared to LS+FT, LS×FT had better outcomes for diagnosing fibrosis stage. However, further studies are required to determine which is the better option for noninvasive diagnosis of fibrosis.

This study is limited in that its design was cross-sectional. It is not clear whether repeated determination of FT score may be useful for tracking the progression of fibrosis and related clinical outcomes, such as occurrence of hepatic decompensation and HCC in individual patients. Therefore, the diagnostic value for predicting the subsequent development of cirrhosis and its various complications with sequential FT measurements during long-term follow-up must be examined further in a longitudinal study. Second, our population did not include patients with F0. As our institute is a tertiary referral hospital and one of the largest medical centers in Korea, patients with relatively more advanced disease status are likely to be referred for close follow-up. It might have resulted in a selection bias and eventually a spectrum bias, since the diagnostic performance of a given noninvasive test tends to increase in general in a cohort with the high disease prevalence and thus the diagnostic performances of LS and FT might have been overestimated in our cohort accordingly. Therefore, another independent external validation study in a population with minimal fibrotic burden should be performed to provide more generalizable results in patients with CHB-related chronic liver disease.

In summary, in a prospective study, we first assessed FT in Asian patients with CHB, demonstrating its comparable diagnostic accuracy to LS for predicting histological fibrosis stage. The optimal suggested cutoff values are expected to be useful as reference values for future studies in Asian patients with CHB. Our results suggest that combined use of LS and FT could avoid invasive liver biopsy in most patients with CHB. We hope that other researchers will evaluate the reproducibility of FT for the noninvasive diagnosis of fibrosis stage in independent populations.
